# Couples’ decision-making on post-partum family planning and antenatal counselling in Uganda: A qualitative study

**DOI:** 10.1371/journal.pone.0251190

**Published:** 2021-05-05

**Authors:** Merlin L. Willcox, Vincent Mubangizi, Silvia Natukunda, Judith Owokuhaisa, Haeven Nahabwe, Florence Nakaggwa, Matthew Laughton, Isabella Chambers, Sabine Coates, Emma King, Emma Fall, Ingrid Muller, Clare Goodhart, Jonathan Graffy

**Affiliations:** 1 School of Primary Care, Population Sciences and Medical Education, University of Southampton, Southampton, United Kingdom; 2 Royal College of General Practitioners, London, United Kingdom; 3 Mbarara University of Science and Technology, Mbarara, Uganda; 4 Bwindi Community Hospital, Kanungu, Uganda; 5 Clarke International University, Kampala, Uganda; 6 University of Bristol Medical School, Bristol, United Kingdom; 7 Department of Public Health and Primary Care, University of Cambridge, Cambridge, United Kingdom; FHI360, UNITED STATES

## Abstract

**Background:**

Although health workers have been trained to provide post-partum family planning (PPFP), uptake remains low in Uganda. An important reason is that women want the agreement of their partner, who is often absent at the time of delivery. In order to address this, we aimed to understand the views of couples and explore barriers and facilitators to implementation of antenatal couples’ counselling on PPFP in Uganda.

**Methods:**

We conducted individual interviews with a purposive sample of 12 postpartum and 3 antenatal couples; and 34 focus groups with a total of 323 participants (68 adolescent women, 83 women aged 20–49, 79 men, 93 health workers) in four contrasting communities (urban and rural) in South-West and Central Uganda. These were recorded, transcribed, translated, and analysed thematically.

**Results:**

Although most participants felt that it is important for partners to discuss family planning, half of the couples were unaware of each other’s views on contraception. Most had similar views on motivation to use family planning but not on preferred contraceptive methods. Most liked the idea of antenatal couples’ counselling on PPFP. The main barrier was the reluctance of men to attend antenatal clinics (ANC) in health facilities. Respondents felt that Village Health Teams (VHTs) could provide initial counselling on PPFP in couples’ homes (with telephone support from health workers, if needed) and encourage men to attend ANC. Suggested facilitators for men to attend ANC included health workers being more welcoming, holding ANC clinics at weekends and “outreach” clinics (in rural villages far from health facilities).

**Conclusion:**

Antenatal couples’ counselling has the potential to facilitate agreement PPFP, but some men are reluctant to attend antenatal clinics. Counselling at home by VHTs as well as simple changes to the organisation of antenatal clinics, could make it possible to deliver antenatal couples’ counselling on PPFP.

## Introduction

Inadequate spacing of births increases the risk of maternal and perinatal mortality and morbidity [1, 2]. Yet 28% of married women in Uganda have an unmet need for contraception, two-thirds of whom have a unmet need for spacing (defined as a gap of less than two years between pregnancies) [3]. As 73% of women in Uganda deliver in a health facility [3], this provides an opportunity for most women to access long-acting reversible contraception (LARC) immediately after delivery, thus addressing their need for spacing [4]. Previous studies have found that immediate postpartum provision of LARC increases use of highly effective contraception at 6 months and improves patient satisfaction [5–7]. Although there have been several projects training health workers to provide post-partum family planning (PPFP), uptake of PPFP and LARCs remains low in Uganda [8, 9]. We conducted a qualitative study in rural South-West Uganda to explore barriers and suggestions for empowering women to access post-partum LARC [10]. One of the main barriers identified by women was the need to get the agreement of their partner before accepting a method of PPFP, although their partners were often absent at the time of delivery. Most women fear taking a decision without their husbands because most men expect to be involved in the decision, and even to be given the final say. Another survey found that 26% of women in Uganda are using contraception covertly [11]. Many women in our study and in others [12] feared that this would lead to relationship problems and violence if discovered. In order to overcome this barrier, participants in our study suggested improving information and counselling for couples on PPFP during antenatal clinics, to enable them to agree on a method of PPFP before the delivery [10]. It was also suggested to combine this with antenatal counselling for HIV testing. Couples’ counselling has the potential to improve concordance in intentions to use family planning, and to reduce covert use, so it is important to understand pre-existing levels of concordance within couples regarding intentions to use family planning. The present study analyses this in greater depth, as well as exploring the feasibility of providing couple’s counselling, through focus group discussions in a wider range of settings in Uganda.

The World Health Organisation now recommends that couples be counselled together prior to testing for HIV, including in antenatal clinics [13]. Uganda adopted this policy in 2006 [14]. The topics to be covered include family planning, in particular in relation to use of condoms in HIV-discordant couples (couples in which one member is HIV positive and the other is HIV negative). A discordant result increases consistent condom use in stable couples [15], and couples’ counselling increases safe sex practices [16]. However, antenatal couples’ counselling would also be useful regardless of HIV status, in planning for immediate PPFP. WHO recommends that antenatal clinics promote the inclusion of husbands and other family members in education and counselling about PPFP [17].

Apart from the specific context of HIV, there has been very little research on antenatal in-person couples’ counselling about family planning, and none of this has focussed on PPFP. Individual antenatal counselling sessions for pregnant women led to improved use of postpartum condoms (but not LARC) in Nigeria [18] and an intervention in Tanzania including individual counselling led to a 6% increase in use of PPIUD (Post-Partum Intra-Uterine Device) [19]. However, a multi-centre study involving South Africa, China and the UK found no impact from counselling women alone [20].

For many health conditions, interventions involving couples are more effective than interventions focussed on an individual [21]. A systematic review of interventions to promote demand for family planning found three studies of counselling for postpartum contraception [22]. The one study of counselling women alone (in Turkey) showed no impact [23]. The other two studies (in Pakistan and Egypt) counselled women together with their husbands, and showed a very significant increase in use of contraception (pooled OR 17.46; 95% CI: 11.32–26.93, P < 0.01) [24, 25].

There has only been one study on couples’ counselling for family planning in sub-Saharan Africa, but it did not focus on postpartum contraception or LARCs. In Malawi, counselling by community health workers (both of individuals and couples) was associated with high levels of uptake of family planning, mainly of the contraceptive injection. However the male partner was only present when the community health worker came to their home in 27% of cases even in the group randomised to receive couples counselling [26]; reasons for this were not recorded. In Ethiopia, an educational intervention on family planning and promotion of discussion between husbands and wives led to a small increase in use of contraception [27]. None of these interventions were based on empirical data about pre-existing knowledge, concerns or levels of discussion between couples. There is a gap in the literature on specific barriers and facilitators to implementing antenatal couple-based counselling on family planning in Africa.

This article aims to provide empirical evidence to inform the design of an antenatal couples’ counselling intervention focussing on increasing uptake of post-partum LARC in Uganda. The specific objectives are:

- To identify the principal factors influencing couples’ communication and decision-making about postpartum LARC, comparing levels of agreement and discordance within couples.
- to understand barriers and facilitators to delivering couples’ counselling in antenatal clinics in Uganda

## Methods

### In-depth interviews

This research took place in two phases. To address the first objective, individual in-depth interviews were conducted with antenatal and postpartum women and their male partners in the catchment area of Bwindi Community Hospital (BCH), Kanungu District, in a rural part of South-West Uganda, in November–December 2015. Potential participants were selected from antenatal clinics and records held by community outreach nurses of women who had delivered within the last six months. Individual interviews took place in a quiet location (usually at the participant’s home, but in a few cases at a health facility); women and men were interviewed separately in the local language (Rukiga). Female participants were interviewed by an experienced female Ugandan qualitative researcher, while their male partners were interviewed by a male Ugandan interviewer, who was trained by the research team and received formative feedback on his interviews. The interviewers had not established any relationship with the interviewees prior to the interviews. Full details are published elsewhere [10], but this analysis focusses primarily on couples in which we were able to interview both the woman and her actual male partner.

### Focus group discussions

After analysis of the in-depth interviews, further funding was obtained to implement and evaluate some of the suggestions made by participants. In particular, we produced health education films on family planning and obtained feedback on these from focus groups in two stages (on the initial version, and on a modified version). One of the messages from the films was the importance of couples discussing and agreeing on a method of family planning. To meet the second and third objectives, we used this opportunity to validate and further explore our findings on couples’ counselling in the focus group discussions. Focus groups were held not only in the same area as the in-depth interviews, but also in three other contrasting areas of Uganda: Mbarara town (South-West), Walukuba (a deprived periurban area in Jinja, South-East Uganda) and Kampala (the capital city). Eighteen focus groups were carried out in June–July 2018 (six in Kanungu district, four in each of the other areas), and a further 16 focus groups in February–March 2019 (four in each area). Before watching the films about family planning, participants were asked about their experiences of discussing family planning with their partner. The films were then shown during the focus groups. After watching the films, they were asked about feasibility and acceptability of various approaches to delivering antenatal couples’ counselling which were suggested by the study team (such as inviting men to attend antenatal clinic with their partner for counselling by a health worker, or counselling at home by VHTs).

In each of the four study areas, we held two separate focus groups, for each of the following population subgroups, to ensure that diverse perspectives were captured:

- Adolescent pregnant or postpartum women attending antenatal or postnatal clinics or baby immunisation clinics.
- Women aged 20–49 attending antenatal or postnatal clinics or baby immunisation clinics at health facilities (whether or not they have partners).
- Men of reproductive age attending the health facility or in the community.
- Health workers (particularly those in charge of antenatal and postnatal clinics, and of family planning services).

The researchers contacted a selection of different types of health centre in each study area and asked the health workers whether they would be willing to take part in the study. The focus group discussions were held at their preferred time so as not to interrupt service delivery.

Potential participants for the other groups were approached by a member of the study team in waiting areas for antenatal, postnatal and immunisation clinics at the same health centres. They were given study information sheets and the study was explained to them. If they wished to continue, they were recruited and consented to be part of the relevant focus group. We purposively sought a range of women of different ages and parities and aimed to have up to 8–12 participants in each focus group, to ensure a wide range of perspectives was represented. We held separate focus group discussions with adolescent girls because they represent a particularly vulnerable group, who may also be reluctant to speak openly in the presence of older women. We recruited men of reproductive age who were married or sexually active, who happened to be visiting the health facility for other reasons, or at meeting places in the community.

Focus groups were held in a quiet place, such as a meeting room, in or near the hospital or health centres from which the participants had been recruited. All focus groups were facilitated in the relevant local languages by experienced Ugandan qualitative researchers, except three of the 10 focus groups with health workers which were facilitated in English by a British male doctor and two British female medical students, who had prior qualitative research experience.

Interviewers and facilitators used semi-structured interview guides (see S1 File), designed for the different types of respondent. Interviews and discussions were audio-recorded on a digital voice recorder, and then transcribed and translated into English. A sample of transcripts were double-checked by an experienced transcriber and where the transcription was inadequate, this was redone.

### Analysis

The transcripts were analysed using thematic analysis [28]. Individual interviews were coded by three researchers (EK, EF and MW) who met to agree on a coding frame. Initial focus groups were coded by three researchers (MW, ML, VM) who also agreed on a coding frame. Coding frames were developed using a combination of deductive and inductive approaches, in order to determine the major themes. When a new theme was identified, we went back to look for it in other interviews and focus groups. We compared the responses of both partners in each couple using a framework approach [29]. This involved creating a table of responses from the male and female partner within each couple, detailing their responses to key questions about discussion with each other, attitudes towards FP and PPFP in particular, preferred method, barriers and facilitators to use of FP. We compared patterns by gender, between clients and health care workers, and looked for emergent patterns. NVivo (Version 11) was used to support the management of analysis. The themes from the in-depth interviews informed the development of the focus group discussion guides, which sought to deepen our understanding of how couples’ counselling might be implemented, and what would be acceptable to a range of different women, men and health workers.

### Ethics

Potential participants were given a participant information sheet, and time to ask questions and consider whether to participate. If they agreed, they were invited to sign a consent form or make a thumb print. In Uganda, pregnant or postpartum women under the age of 18 are considered to be emancipated minors and eligible to provide consent without needing parental consent (UNCST 2014), so the research ethics committees specifically waived the need for parental consent.

The interviewer or facilitator made clear that participants were free not to answer specific questions or to withdraw at any time. Participants in individual interviews were compensated for their time with a small gift worth about 8000 UGX ($2.50) and participants in focus groups were offered refreshments and compensated for their time (10,000 UGX, about $3). Ethical approval for the individual interview study was granted by the Oxford Tropical Research Ethics Committee (OXTREC reference 565–15), from Mbarara University Ethics Committee (ref 11/04-15) and the Uganda National Council of Science and Technology (registration number HS 1922). Approval for the focus groups was granted by the University of Southampton Faculty of Medicine Ethics Committee (Ref:32164); the Bwindi Community Hospital research committee (Ref: BCH/REC/2018); the Mbarara University of Science and Technology Research and Ethics Committee (Ref: MUREC 1/7); the Uganda National Council for Science and Technology (Ref: SS 4615). Administrative approval was granted by all health facilities from which patients and health workers were recruited.

## Results

### Characteristics of participants

Although we identified 26 postpartum women for individual interviews, three could not be found and one refused consent. Of the remaining 22 women, the partner was interviewed in 12 cases (Table 1). An additional three couples were interviewed in the antenatal period. We also interviewed 17 health workers and 18 village health team members (VHTs), but these results are reported elsewhere (10). Eighteen focus groups were carried out in June–July 2018, (total of 173 participants), and a further 16 focus groups in February–March 2019 (150 respondents, Table 2). There was an average of nine participants per focus group, with a range of six to 13.

**Table 1:** Characteristics of the 15 couples in which both partners were interviewed.

Category		Antenatal parent	Postpartum parent	TOTAL
Total		6	24	30
Gender	F	3	12	15
	M	3	12	15
Age group	Teenagers	4	7	11
	20s	2	9	11
	30s	0	5	5
	40 and over	0	3	3
Religion	Catholic	0	5	5
	Protestant	1	9	10
	Pentecostal / “born again”	3	5	8
	Muslim	2	1	3
	Unknown	0	4	4

**Table 2:** Characteristics of focus group participants.

Category	Total	Language version of the film	M	F	Average age
Adolescent women	68	Luganda	0	39	20
		Runyankole	0	29	18
Woman aged 20–49	83	Luganda	0	46	29
		Runyankole	0	37	31
Men	79	Luganda	46	0	36
		Runyankole	33	0	43
Health workers	93	Luganda	7	37	39
		Runyankole	12	37	33

Interview guides contained a question about communication between partners, but the other themes below emerged from the individual interview transcripts: concordance between couples on desire for family planning, on motivations to use family planning, on preferred methods and postpartum contraception. The idea of couples’ counselling emerged from the individual interviews. We asked specific questions about this with a broader range of respondents in the focus groups, to explore different approaches to delivering couples’ counselling (in the community, in health facilities), barriers to antenatal couples’ counselling, and strategies to overcome these. Each of these themes is described below.

### Couples’ communication and decision-making about PPFP

The most consistent theme throughout was the need for joint decision making. The default position is that decision-making power regarding family planning lies primarily with the husbands, who expect their wives to discuss any decisions with them:

Woman: *“Everything I do I have to first ask my husband*. *Recently our husbands came to visit us here in the hospital and they said that if we use family planning without their consent we should not come back home*.*”* (NS18, 19 year old Batwa woman, one of a group of Batwa women staying at the waiting mothers’ hostel, partner of AL07)

Man: *“She can’t do anything without us first talking and we know which method to use*, *is it good for her health or not*.*”* (AL13, 22 year old Mukiga man, partner of NS25)

The dominance of the men resulted in some women simply being too shy to give their opinion, which sometimes led to misunderstandings:

Man: “*I discussed with my wife but she kept quiet*.

*I*: *Why do you think she kept quiet*?

*R*: *I did not understand but she does not like it*.*”* (AL06, 23 year-old man, partner of NS15)

Woman: *“I discussed with him and he said that is what he wants*.

*I*: *Would you please tell me what you discussed*?

*R*: *He said that if I use family planning I will not get pregnant until I finish school*.

*I*: *Do you agree with him*?

*R*: *Yes*.*”* (NS15, 19 year-old woman, partner of AL06)

Women were also scared to use family planning covertly:

“*Men in this village do not like to use family planning and they prohibit their wives from using it*. *So women who come for family planning—they hide from their partners*. *Men know how and where to check especially for those who use implants they know the right arm to check…*. *Men know that the implant is put on the right arm and they know the position so they check their wives to find out whether they went secretly to use family planning*.*”* (NS06, 31 year-old female nurse)

A few men in focus groups said they didn’t mind if their wives used family planning without consulting them:

“*For me I think if my wife can use family planning without consulting me it’s OK because I see that the children that I have are enough… My wife is on family planning but I do not know the method that she is using*. *All I know is that she is on family planning*.*”* (50 year old man, Bwindi focus group)

From the focus group discussions, key factors which enabled men to accept their wives’ decisions on family planning were their education and understanding of family planning, and both partners agreeing that they have had enough children. Men became more accepting after having watched health education films. For example:

“*In the previous periods we as men have not been supporting family planning whereby the women were somehow educated and they knew something about family planning*. *But since the men do not understand much about it*, *they continue discouraging it and you find that since the woman knows about it*, *she goes ahead and uses it without the husbands’ knowledge*. *Through this film*, *I have learnt that sometimes we have to agree*.” (39 year old man, Kampala focus group)

“*Personally*, *my husband is ok with it because we have enough children*.” (30 year old woman, Jinja focus group)

Most focus group participants agreed that it is important for partners to discuss family planning. For example:

*“I think it’s good when one is going to use any family planning method to first discuss it with the husband so that in case of anything he is aware*. *Because the situation can change*. *There are times when people don’t get their periods and others they bleed heavily so I think that it’s better to discuss with the man so that when it has happened he knows why*.*”* (17 year old woman, Mbarara focus group)

#### (a) Concordance on desire for family planning

Apart from one couple, in which neither partner was sure or had thought about family planning, the 15 couples who were interviewed separately could broadly be categorised according to whether the woman and man had the same (concordant) or different (discordant) views on family planning, and according to whether they were aware (conscious) or unaware (unconscious) of their partner’s views (Table 3).

**Table 3:** Summary of levels of agreement between partners regarding family planning, and awareness of their partner’s views (numbers, and example quotes).

	Concordant views (12)	Discordant views (2)
**Aware of partner’s views (8)**	**Consciously concordant (7)** Woman: *“now I want to go back to family planning”* (NS02, 35 year-old postpartum woman, wife of AL02) Man: *“We decided to stop having children”* (AL02, 43 year-old man, husband of NS02).	**Consciously discordant (1)** Woman: *“For me I want it [contraception] but for him he does not want it*.*”* (NS23, 19 year-old woman, Pentecostal, wife of AL12) Man: *“I discussed it with her when she had gone to the health centre for the immunisation of the baby and they told them about Injectaplan*. *When she reached home*, *she told me and found that I didn’t have it in plan*.*”* [meaning his personal plan] (AL12, 23 year old man, Pentecostal, husband of NS23)
**Unaware of partner’s views (6)**	**Unconsciously concordant (5)** *Woman*: *“He just wants us to space well*, *but have more children*, *and yet for me*, *I want to stop having children*.*”* (NS03, 35 year-old woman, partner of AL01) Man: *“I want to stop having children*.*”* (AL01, 36 year-old man, partner of NS03)	**Unconsciously discordant (1)** Woman: *“He told me to use it but I told him I cannot use it when I only have one child so I don’t know what he will decide*?*”* (NS38, 21 year-old mother of one, and wife of AL19) Man: *“She received it well [discussion about family planning]*, *only that we want to go to those big health centres like Bwindi [hospital] because we heard that there is need to first test the blood of a woman to know which method to use so we are waiting for those from Bwindi so that we can enquire from them*.*”* (AL19, 21 year-old husband of NS38)

Most partners agreed with each other, but only about half of couples were consciously concordant–i.e. each partner knew what the other thought and agreed with it. Interestingly there were five couples who thought that the partner had a different view, but in fact they agreed (unconsciously concordant). In four of these five couples, lack of overt agreement meant that the women were not using family planning, although they were hoping to discuss it and start using it if they reached agreement: “*If my husband allows me I can use it*” (NS 21, 18 year old postpartum woman). In only one such couple had the woman covertly obtained contraception. Perhaps her age and the fact that she had already had nine children gave her the confidence to do this:

*Woman*: *“After delivery*, *the nurses advised me to use the tubal ligation method and not to tell my husband about it*. *That is what I did*, *because for him*, *he had told me that he wants me to keep on producing*.*”* (NS29, 40 year-old mother of 9 children, and wife of AL15)

*Man*: *“If family planning would be good and not have the challenges that she has faced previously*, *we would use family planning*.*”* (AL15, 46 year-old husband of NS29)

There were two couples with discordant views, but in only one were both partners aware of their discordant views (consciously discordant). In this couple, the man’s views prevailed and the woman did not use modern family planning although she wanted to; she said she would space her births by avoiding sex during her menstrual periods, and the man said he would use the withdrawal method. In the other couple, the man was unaware that his wife disagreed with his view. This was a young couple who had just had their first child; the man thought his wife wanted family planning, whereas she did not because she feared side-effects. In this couple the woman’s preference had so far prevailed–she had not started using any family planning although she had delivered 2 months previously and wanted to wait 2 years for her next pregnancy.

Disagreements and distrust can have serious consequences. One woman (whose partner was not interviewed) reported how her husband had taken a second wife because he suspected her of using family planning and he wanted to have more children:

“*He asked me why I want to use family planning when I have only 3 children*. *When I lost my baby I was not able to conceive for some time and he suspected that I was using family planning and so he married another wife*.

*I*: *Did you truly use family planning that time*?

*R*: *No I did not*. *It just happened*, *but for him*, *he thought that I had used family planning*. *He even told me that if I do not want to produce children*, *he will marry*, *so he married another wife*. *He says he was born as one child so he wants to have many children*.*”* (NS04, 25 year-old Catholic woman)

#### (b) Concordance on motivation to use family planning

In general, most couples agreed on the two main reasons for using family planning: limiting family size (because of limited land, food and money for schooling) and spacing children. One woman said she would not want to have another child if the process of giving birth was difficult, and one man mentioned that he needed to get himself “organised” before going back to work in Kampala.

*“We have a small piece of land for digging to get food for our children*. *Our children get infected by worms often because they do not get enough food and we ran up and down to get milk for them so that is why he wants us to stop on the number of children that we have by using family planning”* (NS03, 35 year-old postpartum woman, partner of AL01)

*“We had our first child followed immediately and we had challenges up bringing this child so we learnt our lesson well”* (AL04, 34 year-old man, partner of NS12)

*“if she gets pregnant and I leave her here alone and I go back to Kampala*, *she may suffer without anyone to help her*, *because I have not settled well at my work place*. *So as to take her there*, *so I want us to first be organized and talk about it*.*”* (AL06, 23 year-old man, partner of NS15)

#### (c) Concordance on preferred methods of contraception

Overall, there was broad agreement that the injection was the most popular method both among men (7) and women (6), because this was the most familiar method for them:

“I: Why have you chosen the Injectaplan as the method your wife should use?

*R*: *It is the one I want her to use*.

*I*: *Are there any methods that you wouldn’t want your wife to use*?

*R*: *That is the only method I know*.*”* (AL22, 19 year-old man, husband of NS41)

The implant was the second most popular method for men (3) and women (4). However, levels of agreement were very low between partners in the individually interviewed couples on the preferred method of contraception. In only one couple did both partners prefer the implant, and in only three couples did both partners prefer the injection. In all the other couples, each partner expressed a different preference. Only one man and one woman (not in the same couple), chose the IUD as their preferred method. This suggests that few couples had discussed which method to adopt, and yet it was felt important for couples to do this and to agree on a specific method to use:

*“I don’t think they don’t want to use Injectaplan or IUD but I think both a man and woman sit and agree that*, *“let us use this method*. *If it doesn’t work let us leave it and use this other method”—until they find a better method for themselves*.*”* (AL17, 28 year-old man, partner of NS34)

As part of this discussion, it is also important for both partners to be aware of possible side effects. Men felt they would be more confident about uptake of contraceptives, if they had first heard from a health professional about the side effects their partner may experience and how to manage these.

*“Some of the mothers say get some weaknesses*, *like somebody will become—always will feel fatigue*. *Some of them gain much weight*, *they develop let’s say hypertension or whatever*, *you find they cannot even dig*, *they cannot do what—because of all those few side effects*. *If they were not heard by the man*, *then the man may not be supportive*.*”* (MW04, male community outreach nurse)

*“if I come with her*, *I can understand what she is going to use*, *when we shall have children and the side effects that she may go through*, *even me I understand so I can’t wonder the cause of the problem”* (P12–24 year-old man, Walukuba focus group)

#### (d) Concordance on postpartum contraception

Most men saw the advantage of immediate post-partum contraception when they understood that a woman can become pregnant even before 6 weeks after the delivery:

*“I think it is a good thing because I have taken long without being with a woman so if she comes home without a method we may both agree to have sex and I make her pregnant”* (AL08, 19 year-old man, partner of NS17)

In contrast almost all women preferred to wait until 6 weeks after delivery. Only one woman, a VHT, remarked that early IUD use would be necessary if a husband was not willing to abstain for 6 weeks:

*“I think if one has a cooperative man she can insert after 6 weeks but if one has a man who does not cooperate with her she should use it immediately because it does not pain a lot if inserted immediately after delivery*.*”* (NS36, 50 year-old female VHT)

Few couples had discussed the possibility of receiving contraception immediately after delivery. This was an important barrier to PPFP, since men expect to be consulted but few are present at antenatal visits or around the time of delivery.

“*I*: *What do you think about use of family planning for women who have recently delivered before they are discharged*?

*R*: *It’s bad*.

*I*: *Why*?

R: Now you see at times they can discharge you when your husband is not around and so you cannot just use family planning without his consent. In our family our men are very complicated: he may even cut off your arm!”

(NS21, 18 year-old woman, partner of AL11)

A comprehensive analysis of fears, side-effects experienced and perceived barriers to the use of PPFP and LARC has been reported previously (10). There was a high level of concordance within couples regarding these fears, especially that women needed time to recover from the delivery before using contraception, particularly if they were bleeding.

“*R*: *It can be said even on the radios—there is one who said that a woman died due to use of an implant*.

*I*: *How would you feel if your wife used this method immediately after giving birth*?

*R*: *I would give her strict restrictions of not over working herself*, *not digging for long hours*, *do little work*.*” (AL11*, *20 year-old husband of NS21)*

*“Women who are still bleeding should not use it because they may bleed a lot since they may not have recovered from child birth*.*”* (NS25, 19 year-old woman, wife of AL13)

Women were more likely to fear that the side-effects such as bleeding and loss of libido would lead to relationship difficulties or even the break-up of their marriage. After these fears had been successfully addressed by health education films, participants in focus groups were asked whether they could discuss postpartum contraception with their partner by telephone if he was not present after the delivery. Neither women nor men seemed happy with this:

*“With phone calls*, *since he is far from you*, *he can even deny that you discussed about it…*. *You can even run out of credit while explaining the implant*.*”* (P5, 20-year old woman, Walukuba focus group)

*“I believe that she can come to the hospital for another time for any case*, *so if she comes back and you discuss about it and she convinces you well*, *then you can decide to use it*. *But on phone*, *it is a limited time*.*”* (P5, 33 year-old married man, Walukuba focus group)

### Couples counselling for family planning

In order to facilitate communication between partners and overcome these barriers to uptake of PPFP, the main suggestion emerging from the analysis of couples’ interviews was couples counselling. The focus groups confirmed that this would be useful: receiving the information together would mean that both partners would have a better understanding of the options available.

“*I think it is good because when we go together*, *we get to know the danger of having many children and we can agree with our wives on what to do to have few children*.*”* (P5–44 year-old man, Bwindi focus group)

Health workers also felt that couples’ counselling would lead to better communication of family planning views, would reduce conflict, and lead to a well informed decision.

*“I also think [couples counselling] is a good idea because … they are able to make an informed decision on which type of family planning to use that is convenient for both of them*. *For example if they are to use the implant and some side effects come up*, *so the man will bear with the woman if such side effects are happening*. *He will have known before and so he will not bother the woman”* (P4–36 year-old midwife, Mbarara focus group)

However, some health workers felt that a single episode of couples counselling may not be sufficient, because men can change their minds. They would want to have seen the man several times before feeling comfortable to provide post-partum contraception to the woman, in the absence of her partner:

“***P2*** *–The father might agree in front of a nurse or a midwife*. *But after delivery you will never see him again and he can’t allow the mother to take the method*.

***P1** –they keep changing their minds*.

**Facilitator**–So if they agreed in the antenatal clinic that the mother could have, immediately after delivering, she could have the implant or the IUD, would you then be able to give that after she delivers here?

***P1—**Yes*.

**Facilitator**–Or would you again need to find the husband a second time?

***P1 –****we would give*. *The only thing we would give post-partum first line is the IUD*. *That one we can give*. *If we are sure that the mother has been talking with the husband throughout antenatal*, *not only at one visit but throughout*, *then we can give*.*”* (Focus group of health workers, Mpungu Health Centre III)

#### (a) Barrier: Men are reluctant to attend ANC

The focus groups revealed several barriers to men attending ANC. Some husbands worked away from home and felt they didn’t have time to attend. Others could not afford the cost of transport for an extra person to go to the health centre. But a more fundamental reason was that many husbands didn’t see the need for them to attend ANC:

*“For me I have not been escorting my wife to the health centre because I do not see the need of doing that*. *I think women are the ones who know what they do there”* (P8, 48 year-old man, Mbarara focus group)

It also emerged that some men felt stigmatised for attending the antenatal clinic with their partner. In several sites, some men (especially younger men) mentioned that they felt ashamed or shy to accompany their wife:

*“We went together for family planning like the way you are telling us that would we wish to go together*, *and they taught us*. *That is the reason as to why I cannot go with her because I got ashamed since I was the one who took her so I don’t want to become ashamed again*.*”* (P10, 25 year-old man, Walukuba focus group)

“*P2*: *The reason is this*: *he can reach there and he is asked many questions and yet he is shy to answer them so he sends you there alone*. *Most of the men you can look at his face and think that he can talk but when he is in amidst people he fails to talk*.

*P1*: *They lack self-esteem and most of them are still young*. *Another one can be around*, *but when he is busy he cannot come*. *Something else is that we get married when we are still young and we get young men- if probably they were mature*, *you would tell them and they come*.

*P4*: *Most men just lack self-esteem*.” (Adolescent women, Mbarara focus group)

Aside from this, the long time taken queuing was seen as a major barrier:

“*the large lines of people at the health facilities—where one can even spend the whole day waiting for services—makes men discouraged*, *because they see it as time wastage*.*”* (P5, 51 year old nurse, Mbarara focus group)

*“My wife normally tells me to escort her but I don’t have that time*. *They delay there a lot and she tells me there is always a long line”* (P8, 48 year old man, Mbarara focus group)

#### (b) Possible facilitator: Combination with antenatal HIV counselling and testing

Some individual interviewees suggested that there would be scope to combine couples’ counselling on family planning with couples’ counselling on HIV testing, during the Antenatal Clinic (ANC):

*“R: I think women should discuss with their husbands and they agree and go to the health centre without any fear and be confident of what they want*.

*I*: *Did you go with your wife when she was going for antenatal*?

*R*: *No I didn’t*.

*I*: *How would you feel if you went with her*?

*R*: *I think I would feel good because both of you can test for HIV and know how your lives are*.*”* (AL11, 20 year-old man, partner of NS21)

However, many people in focus groups felt that HIV testing would deter some couples from attending ANC together. They feared they would have discordant results and didn’t want their partners to discover this:

*“Most of the men cheat on their wives so they fear that their wives are going to find out when they test positive*, *or even they fear that their marriages will break if the woman turns to be positive—and the dilemma of explaining the discordance—and of course the blame game*.*”* (P5, 45 year-old woman, Bwindi focus group)

*“For me I think even us women we hide our husbands our return dates because we also fear that we could be the ones with HIV and so we fear that blame game of who brought the HIV into the family*. *So some women do not ask their husbands to escort them to the hospital*.*”* (P1, Adolescent woman, Bwindi focus group)

*“Even I hear they test for HIV—so for me*, *I don’t want to test for HIV with my wife*. *I can test alone and I tell her the results*.” (P8, 48 year-old man, Mbarara focus group)

However, this was not the case for all men and some felt it would be better to test with their partner, as this would lead to a greater sense of unity and involvement as a couple:

*“I do not agree with that*, *I think it is good that we both test because when you test together*, *your wife can trust you more and if you are both sick then still you begin medication together”* (P5, 48 year-old man, Mbarara focus group)

Some health workers also felt that it would be better for couples to receive their HIV results together, so they could be given effective counselling and to minimise the impact that the disclosure may have upon them. Some felt they would have time to do this (because currently few couples come together) but there is a potential opportunity cost in that others who are kept waiting may leave before receiving any antenatal care. Therefore if demand for couples counselling increased, there would need to be more staff to provide this.

*“It would be very good if one receives information on both*. *I may not test that same day but I will come back for the test*, *you can talk about it and those who feel they can*, *they do—rather than denying the chance*.*”* (P2, 28 year-old male doctor, Mbarara focus group)

*“Remember you have to work through like 40 clients in the day and even when you are giving those comprehensive services at once then*, *take an example*, *you get like 10 couples and you need to do that comprehensive counselling—so it takes time and sometimes you are finding clients are disappearing before they receive the services”*. (25 year old midwife, Bwindi focus group)

The very limited time available in busy antenatal clinics was a concern for both service users and health workers. Service users felt that complete information and advice was not always provided:

*“The nurses are busy*, *and they do not give pre-test and post-test counselling*, *even for those that turn out to be positive they do not give them time to have good couple counselling*.*”* (P1, 52 year-old man, Bwindi focus group)

*“You can’t really elaborate HIV then you go to family planning*. *There are other issues of antenatal also so all what we do is just hinting on the major things for each topic*. *You hint on everything because they have not come for family planning*, *they have come for antenatal majorly*. *They are interested the other*, *but you are also bringing in this one which you think that it is important*. *You can’t have enough time to talk about everything*.*”* (P8, 28 year old midwife, Walukuba focus group)

#### (c) Possible facilitator: Reducing waiting times for couples

In order to reduce the time men spend queuing, health workers in most areas described giving priority to women who attend the ANC with their husbands, as an incentive to encourage them and to set an example for others:

*“We are encouraging them*. *Even when they come*, *we prioritize*. *Those who have come with their husbands*, *we give them first priority because the men are ever impatient*, *so we want to serve them quickly and they go*.*”* (P5, 47 year-old female midwife, Walukuba focus group)

Interestingly, there were reports of some women subverting this process by paying another man to accompany her in order to avoid queuing:

*“It is good to give the priority to couples but you have to be careful*, *because there is a hospital I was in and they knew that when you go with a man*, *you are given the first priority—so they would go with boda-boda men*, *pay them to escort them each time their husbands refused to come with them*.*”* (P6, 22 year old nurse, Bwindi focus group)

#### (d) Possible facilitator: Politeness

Women felt it especially important that health workers should be polite to their husbands, which unfortunately is not always the case:

*“There are some nurses who are so strict and a man comes with the wife and she is not in a good state because they don’t have money and they speak rudely to the man asking him many questions*. *He will hate it and never come back*. *But when the man comes and you explain to him nicely why he should keep coming*, *he will always come with the wife*.*”* (P4–18 year-old woman, Mbarara focus group)

#### (e) Possible facilitator: Weekend clinics

Timing of antenatal clinics was felt to be important in urban areas where most men work during the week and are more available at weekends. However, at a focus group in a rural area, health workers reported that although they already hold ANC on Saturdays, they did not receive more men then. Health workers agreed they could work on Saturdays provided they were paid extra.

*“Some men claim to be very busy even when on some days they are free; the usual free days are over the weekends like Saturdays and yet the antenatal clinics do not operate on those weekends*. *If it were Saturday*, *some men are free*, *however*, *weekdays are difficult*.*”* (P1–31 year-old woman, Walukuba focus group)

*“that would be extra work and it needs to be paid a side allowance*.*”* (P5, 51 year old nurse, Mbarara focus group)

#### (f) Possible facilitator: Outreach clinics by health workers

Mobile outreach antenatal clinics in the community were suggested as a way of addressing the challenge of couples needing to travel long distances to health facilities in rural areas:

*“I think they can put outreaches in the villages for ANC to avoid the issue of transport”* (P4, 60-year old man, Bwindi focus group)

However, in one area health workers were already doing community visits and still reported difficulties in reaching men:

*“men are busy*, *so when you visit 10 households*, *you will be able to meet like two men there and you can talk to those two*, *but miss out on the 8 men*.*”* (P2, 31- year old male community nurse, Bwindi focus group)

Another suggestion for overcoming the travel barrier was to reimburse men for their travel costs to attend antenatal clinics:

*“Some of us men do not come because we are busy but I was thinking if the health workers give us some incentives to encourage us to come*, *we can try and come*. *For example if they give us money for transport*, *we can come with our wives to get the couple counselling and testing*.*”* (P5–48 year-old man, Mbarara focus group)

#### (g) Possible facilitator: Counselling by community health volunteers

Many couples in villages first consult community health volunteers (known in Uganda as Village Health Teams or VHTs), who are probably the most accessible for providing initial counselling to couples:

*“Most importantly is agreeing with a husband and then go to the VHT for advice*.*”* (P5 –Adolescent woman, Bwindi focus group)

However some participants, including VHTs themselves, felt this needed to be followed up in health facilities (because VHTs often have very low levels of training; a few are able to provide basic contraceptives, but none can provide LARCs).

*“Women should come with their husbands to the hospital and you meet them together and explain to them about the methods*, *even we have already taught them as VHTs*, *so that they can choose the method together with their husbands*.*”* (AL09, 46 year-old male VHT)

*“The health worker would be better because she is the one who has most of the information and who performs the process of inserting*.*”* (P9, Man, Bwindi focus group)

Likewise, health workers felt that Village Health Teams (VHTs) have an important role in encouraging men to attend antenatal counselling in health facilities, as they felt they may not be able to answer all the questions about family planning:

“I—How do you think we could encourage men to come to couples counselling?

*P2 –I think that one will be done through community sensitisation—by using VHTs to target the communities for sensitisation*.*”* (P2–40 year-old female midwife, Bwindi focus group)

*“I think the VHTs*, *when given information*, *they can*, *but they may not be able to answer some questions which are technical*!*”* (P7, registered nurse, Kampala)

If the man did not want to come to the health facility, but had questions which the VHT did not feel confident in answering, another potential solution was for the VHT to phone the health worker so the couple could speak to them by phone. All focus groups were happy with this suggestion; only one health worker expressed reservations that the community may lose trust in the VHT.

*“VHT can come alone—it is okay—and if he has a phone*, *so we can ask questions where need be to the health worker”* (Women’s focus group, Bwindi)

## Discussion

### Summary and contextualisation of main findings

Although most couples had concordant attitudes towards family planning, many were not aware of their partner’s views. There were a few who disagreed with each other. Few couples had specific plans or knowledge of the contraceptive options available to them, which suggests a lack of detailed discussion between partners. A recent quantitative survey in Uganda showed that 17.9% of women had not discussed the decision to avoid pregnancy with their partner prior to using a contraceptive; 41.8% of them discontinued the contraception (as opposed to 28.2% of those who had discussed it) and this was the most predictive factor for discontinuation [30]. Our qualitative study provided detailed suggestions as to how such a discussion could be encouraged and facilitated.

Several individuals suggested couples’ counselling and focus groups confirmed that people liked this idea. Two studies of couples’ counselling have shown that this can improve uptake of PPFP: three hour-long sessions in Egypt [25] and one 20-minute session in Pakistan [24]. Couples’ counselling has been tested extensively in the context of Couples HIV Testing and Counselling (CHTC), which involves a couple coming together to be tested for HIV at the same time and receiving the results together. CHTC has been associated with increased communication between couple members, sexual behaviour change, and decreased HIV transmission [16, 31]. Both the World Health Organization (WHO) and the Ugandan government have recommended that CHTC be offered wherever HIV testing and counselling are already available (including antenatal clinics).

Our study has shown that the main barriers to implementing antenatal couples’ counselling were about time, location, cultural stigma of men attending ANC and inclusion of HIV testing. A qualitative study in Eastern Uganda also found that uptake of antenatal couples’ counselling for HIV testing remains low because men often feel unwelcome or treated rudely at health facilities: they are asked to wait outside while their partners are attended to during antenatal clinics. This study also found that men feared marital conflict if the testing were to reveal that they were HIV positive [14]; many men felt that their marriages were distrustful and unstable, and so were unlikely to agree to HIV testing with their partners; only those in a loving and caring relationship were willing to test together [14]. This was confirmed by the very mixed reactions from our focus groups. The same study suggested that it will be important to make health facilities less stigmatizing and more male-friendly, offering more flexible opening hours [14]–this is also supported by our findings.

Other suggestions from our participants for overcoming barriers included outreach ANC in villages, reducing waiting times for couples, and VHTs providing initial counselling. Several of these had been tried in some places with varying degrees of success, and some require extra funding. The idea with the most support seemed to be VHTs counselling couples in their homes, encouraging them to attend ANC together, but with the option to phone a health worker when extra advice was needed and the man was not prepared to attend ANC with his partner. A previous randomised controlled trial of prenatal counselling on PPFP by VHTs showed no impact on uptake of postpartum contraception in Uganda [32]. However, this counselling did not involve husbands in receiving the counselling, or health workers in delivering counselling. Our research suggests that future studies should investigate the effectiveness of counselling both partners in a couple together, involving not only VHTs but also involving health workers.

### Strengths and weaknesses

To our knowledge, this is the first qualitative study comparing the views of men and women within couples regarding post-partum family planning in Uganda, and the first to explore views on antenatal couples’ counselling on family planning. A strength of the work is that partners were interviewed independently. It is possible that our sample of individual interviews was biased towards couples in which both partners agreed on PPFP–because those who declined to participate may have been more likely to hold discordant views. However, findings were confirmed in a large sample of focus groups in several different parts of Uganda, covering a wide range of people in a variety of urban and rural settings, and including three major ethnic groups. The only major difference which emerged between urban and rural settings was the suggestion to hold antenatal clinics on Saturdays (in urban areas) and to hold outreach clinics in villages (in rural areas).

### Implications for programme design

It is important to be aware that in many Ugandan couples, partners may not be fully aware of each other’s views on PPFP; there may also be disagreements. These can lead to covert contraception use, which in turn may result in threats or violence if the use is discovered [12] and is associated with double the risk of discontinuation [30]. Counselling during the antenatal clinic will avoid the situation of women having obtained contraception covertly (because all of the women are already pregnant) and is an opportunity to involve both partners in the discussion. Most participants had only superficial knowledge of the available methods, suggesting that counselling should improve the provision of accurate information about these. There was general agreement that initial couples’ counselling could be conducted by VHTs in the patients’ homes, but that this should be followed up by a health worker (either in person, or by telephone). In order to overcome men’s reluctance to attend ANC, it is important that health workers are polite to them and reduce waiting times. If resources allow, other strategies could be tried such as providing extra clinics at weekends and outreach to remote areas, and combining with HIV counselling and testing.

### Unanswered questions and priorities for future research

Operational research is needed to assess how antenatal couples’ counselling can best be implemented in Uganda. A pilot study is needed to answer a number of feasibility questions which are important in designing a couples’ counselling intervention. In particular, it is important to assess whether VHTs and health workers can be trained to provide respectful, effective counselling to couples; whether it is possible to do this in outreach visits or extra weekend clinics; whether this will enable couples to reach common agreement on PPFP antenatally and whether it is possible to achieve this after a single session. It will be important to quantify how many couples will attend ANC together, and how many will change their minds at the time of delivery. It will also be important to conduct a qualitative evaluation, in particular to understand whether there could be any unintended negative effects, for example whether inclusion of men in antenatal counselling may disempower women or reduce their autonomy, or whether prioritisation of couples attending ANC together may create inequities in quality of care for other women. For couples who do not attend ANC together, it will be interesting to evaluate whether counselling by a VHT would be adequate; telephone support could be provided by a health worker for the VHT, in case of questions they cannot answer. It would be useful to investigate whether counselling on PPFP can be combined with optional pre-test counselling for HIV testing, without deterring men from attending. It will also be important to assess whether VHTs can effectively follow up women who opt to receive contraception at 6 weeks postpartum or when their baby is immunised, how many of these actually take up PPFP and which intervention components are most cost-effective for reducing unmet need for contraception. If an antenatal couples’ counselling intervention proves feasible, the next stage would be to conduct a randomised controlled trial, to assess its effectiveness and cost-effectiveness to reduce unmet need for PPFP.

## Conclusions

Agreement of both partners in a couple is an essential pre-requisite for most women to accept PPFP in Uganda. If this has not been discussed prior to delivery, most women will refuse PPFP until they have the agreement of their partner. Yet this study shows that couples often have not come to an explicit agreement on use of PPFP and indeed hold divergent views on which method they prefer. Couples may find it difficult to agree by themselves on the most appropriate method and timing of PPFP for their particular circumstances. Facilitation of a discussion on this topic, first by VHTs and then by health workers, has the potential to overcome this problem. Couples’ counselling on PPFP should explore the views of each partner on their motivation to use family planning, their preferred timing for PPFP, the menu of available methods, their advantages and possible side-effects. Further research is needed to assess the feasibility of implementing couples’ counselling on PPFP during antenatal clinics in Uganda.

## Supporting information

S1 File Interview and focus group discussion guides. (DOCX)

## References

[1] Conde-Agudelo A, Belizán JM. Maternal morbidity and mortality associated with interpregnancy interval: cross sectional study. British Medical Journal. 2000;321(7271):1255–9. 10.1136/bmj.321.7271.1255 11082085 PMC27528

[2] Conde-Agudelo A, Rosas-Bermudez A, Kafury-Goeta AC. Birth spacing and risk of adverse perinatal outcomes: a meta-analysis. Journal of the American Medical Association. 2006;295(15):1809–23. 10.1001/jama.295.15.1809 16622143

[3] Uganda Bureau of Statistics—UBOS, ICF. Uganda Demographic and Health Survey 2016. Kampala, Uganda: UBOS and ICF; 2018.

[4] Royal College of Obstetricians and Gynaecologists. Best practice in postpartum family planning. London: Royal College of Obstetricians and Gynaecologists; 2015.

[5] Averbach S, Kakaire O, Kayiga H, Lester F, Sokoloff A, Byamugisha J, et al. Immediate versus delayed postpartum use of levonorgestrel contraceptive implants: a randomized controlled trial in Uganda. American Journal of Obstetrics & Gynecology. 2017;217(5):568.e1–.e7. 10.1016/j.ajog.2017.06.005 28610898 PMC6310612

[6] Lester F, Kakaire O, Byamugisha J, Averbach S, Fortin J, Maurer R, et al. Intracesarean insertion of the Copper T380A versus 6 weeks postcesarean: a randomized clinical trial. Contraception. 2015;91(3):198–203. 10.1016/j.contraception.2014.12.002 25499587

[7] Lopez LM, Bernholc A, Hubacher D, Stuart G, Van Vliet HA. Immediate postpartum insertion of intrauterine device for contraception. Cochrane Database of Systematic Reviews. 2015;6:CD003036. 10.1002/14651858.CD003036.pub3 PMC10777269 26115018

[8] Uganda Bureau of Statistics, ICF International Inc. Uganda Demographic and Health Survey 2016: Key Indicators Report Kampala, Uganda and Rockville, Maryland, USA: Uganda Bureau of Statistics and ICF International; 2017 [Available from: http://www.ubos.org/2017/03/15/uganda-demographic-and-health-survey-2016-key-indicators-report/.

[9] Sileo KM, Wanyenze RK, Lule H, Kiene SM. Determinants of family planning service uptake and use of contraceptives among postpartum women in rural Uganda. International Journal of Public Health. 2015;60:987–97. 10.1007/s00038-015-0683-x 25967466 PMC4644123

[10] Willcox M, King E, Fall E, Mubangizi V, Nkalubo J, Natukunda S, et al. Barriers to Uptake of Postpartum Long-Acting Reversible Contraception: Qualitative Study of the Perspectives of Ugandan Health Workers and Potential Clients. Studies in Family Planning. 2019;50(2):159–78. 10.1111/sifp.12088 30963601

[11] Heck CJ, Grilo SA, Song X, Lutalo T, Nakyanjo N, Santelli JS. "It is my business": A Mixed-Methods Analysis of Covert Contraceptive Use among Women in Rakai, Uganda. Contraception. 2018;98(1):41–6. 10.1016/j.contraception.2018.02.017 29514043 PMC6041694

[12] Kibira SPS, Karp C, Wood SN, Desta S, Galadanci H, Makumbi FE, et al. Covert use of contraception in three sub-Saharan African countries: a qualitative exploration of motivations and challenges. BMC Public Health. 2020;20(1):865. 10.1186/s12889-020-08977-y 32503485 PMC7275340

[13] World Health Organisation. Guidance on couples HIV testing and counselling including antiretroviral therapy for treatment and prevention in serodiscordant couples: recommendations for a public health approach. Geneva: World Health Organization; 2012. 23700649

[14] Larsson EC, Thorson A, Nsabagasani X, Namusoko S, Popenoe R, Ekström AM. Mistrust in marriage-Reasons why men do not accept couple HIV testing during antenatal care- a qualitative study in eastern Uganda. BMC Public Health. 2010;10(1):769. 21167040 10.1186/1471-2458-10-769 PMC3018443

[15] Rosenberg NE, Pettifor AE, De Bruyn G, Westreich D, Delany-Moretlwe S, Behets F, et al. HIV testing and counseling leads to immediate consistent condom use among South African stable HIV-discordant couples. Journal of acquired immune deficiency syndromes (1999). 2013;62(2):226–33. 10.1097/QAI.0b013e31827971ca 23117500 PMC3548982

[16] Rosenberg NE, Graybill LA, Wesevich A, McGrath N, Golin CE, Maman S, et al. The Impact of Couple HIV Testing and Counseling on Consistent Condom Use Among Pregnant Women and Their Male Partners: An Observational Study. Journal of acquired immune deficiency syndromes (1999). 2017;75(4):417–25. 10.1097/QAI.0000000000001398 28426440 PMC5493523

[17] World Health Organisation. Programming strategies for postpartum family planning. Geneva: World Health Organisation; 2013.

[18] Adanikin AI, Onwudiegwu U, Loto OM. Influence of multiple antenatal counselling sessions on modern contraceptive uptake in Nigeria. The European Journal of Contraception & Reproductive Health Care. 2013;18(5):381–7. 10.3109/13625187.2013.816672 23885659

[19] Pearson E, Senderowicz L, Pradhan E, Francis J, Muganyizi P, Shah I, et al. Effect of a postpartum family planning intervention on postpartum intrauterine device counseling and choice: evidence from a cluster-randomized trial in Tanzania. BMC women’s health. 2020;20(1):102–. 10.1186/s12905-020-00956-0 32398077 PMC7218519

[20] Smith KB, van der Spuy ZM, Cheng L, Elton R, Glasier AF. Is postpartum contraceptive advice given antenatally of value?☆. Contraception. 2002;65(3):237–43. 10.1016/s0010-7824(01)00308-0 11929646

[21] Arden-Close E, McGrath N. Health behaviour change interventions for couples: A systematic review. British journal of health psychology. 2017;22(2):215–37. 10.1111/bjhp.12227 28150410 PMC5408388

[22] Belaid L, Dumont A, Chaillet N, Zertal A, De Brouwere V, Hounton S, et al. Effectiveness of demand generation interventions on use of modern contraceptives in low- and middle-income countries. Tropical Medicine and International Health. 2016. 10.1111/tmi.12758 27465589

[23] Akman M, Tüzün S, Uzuner A, Başgul A, Kavak Z. The Influence of Prenatal Counselling on Postpartum Contraceptive Choice. Journal of International Medical Research. 2010;38(4):1243–9. 10.1177/147323001003800405 20925996

[24] Saeed GA, Fakhar S, Rahim F, Tabassum S. Change in trend of contraceptive uptake—effect of educational leaflets and counseling. Contraception. 2008;77(5):377–81. 10.1016/j.contraception.2008.01.011 18402856

[25] Soliman M. Impact of antenatal counselling on couples’ knowledge and practice of contraception in Mansoura, Egypt. East Mediterr Health J. 1999;5(5):1002–13. 10983541

[26] Lemani C, Tang JH, Kopp D, Phiri B, Kumvula C, Chikosi L, et al. Contraceptive uptake after training community health workers in couples counseling: A cluster randomized trial. PLOS ONE. 2017;12(4):e0175879. 10.1371/journal.pone.0175879 28448502 PMC5407810

[27] Tilahun T, Coene G, Temmerman M, Degomme O. Couple based family planning education: changes in male involvement and contraceptive use among married couples in Jimma Zone, Ethiopia. BMC Public Health. 2015;15:682. 10.1186/s12889-015-2057-y 26194476 PMC4509724

[28] Braun V, Clarke V. Using thematic analysis in psychology. Qualitative Research in Psychology. 2006;3(2):77–101.

[29] Ritchie J, Spencer L. Qualitative data analysis for applied policy research. In: Bryman A, Burgess RG, editors. Analyzing qualitative data. London: Routledge; 1994. p. 173–94.

[30] Sarnak DO, Wood SN, Zimmerman LA, Karp C, Makumbi F, Kibira SPS, et al. The role of partner influence in contraceptive adoption, discontinuation, and switching in a nationally representative cohort of Ugandan women. PLOS ONE. 2021;16(1):e0238662. 10.1371/journal.pone.0238662 33434205 PMC7802956

[31] Kennedy CE, Medley AM, Sweat MD, O’Reilly KR. Behavioural interventions for HIV positive prevention in developing countries: a systematic review and meta-analysis. Bulletin of the World Health Organization. 2010;88(8):615–23. 10.2471/BLT.09.068213 20680127 PMC2908966

[32] Ayiasi RM, Muhumuza C, Bukenya J, Orach CG. The effect of prenatal counselling on postpartum family planning use among early postpartum women in Masindi and Kiryandongo districts, Uganda. The Pan African Medical Journal. 2015;21:138. 10.11604/pamj.2015.21.138.7026 26327975 PMC4546801

