# Supplementary material for: Couples’ decision-making on post-partum family planning and antenatal counselling in Uganda: A qualitative study
Source: PLoS One. 2021 May 5;16(5):e0251190. doi: 10.1371/journal.pone.0251190 (PMC8099118; doi:10.1371/journal.pone.0251190)
Supplement: S1 File — (DOCX) [file pone.0251190.s001.docx]

PONE-D-20-10104
Exploring barriers and facilitators to antenatal couples’ counselling on contraception in Uganda: a qualitative study

# S1 File: Interview and Focus Group Discussion Guides

## Interview Schedule for Postpartum Women

**Version 1, 28^th^ April 2015 OXTREC Reference 565-15**

Participant Identification number: _____ Partner’s identification number: _____

Opportunity to ask questions and sign consent form

*Read out participant information sheet/get them to read it if literate.*

- Do you have any further questions?
- Would you like to talk to me about family planning?
- Do you understand and agree with everything I have said?

*If yes: Read consent form with participant and ask to sign or make a thumb print*

- Is it ok to start the interview now?
- I am going to turn on the tape recorder now and we can start.

Interview

1. Can you tell me a bit about yourself and your family?

*Probes:*

- Age, occupation, religion, education level, ethnic group
- Tell me about your partner. Married/unmarried, live together/apart, length of relationship, ethnic group of partner
- How many pregnancies have you had? How many children do you have? Have you lost any children?
- Do you want to have another child?
  - If yes, how long would you like to wait?

1. Have you and your partner discussed family planning recently?

*Probes:*

- What did you discuss?
- When did you discuss this?
- What do you think he thinks about family planning? Is this the same or different to what you think about family planning?

1. Have you used family planning?

*Probes if yes:*

- What methods of family planning have you used?
- When did you use it?
- What was your experience using it?

*Probes if no:*

- Have you ever discussed family planning with a health worker?
- Have you ever thought of using family planning?
- What methods do you think might suit your needs?

1. Were you offered family planning after this delivery or after previous deliveries? Can you tell us your experience?

*Probes:*

- Did you discuss family planning with your midwife or nurse?
- Did you attend post-natal clinic? What was your experience then?
- Did you speak to your VHT about family planning? Did you discuss it while you were pregnant or since delivery?

*Further probes* ***if offered*** *family planning:*

- What did you think about being offered family planning at this time?
- Did you decide to take up family planning? Why/why not?
- What method did you choose? Why?
- Are there any methods you would avoid? Why?

*Further probes* ***if not offered*** *family planning:*

- Would you have liked to discuss family planning with a health worker after your delivery? Why/why not?
- If family planning had been offered to you, do you think you would have taken it up? Why/why not?
- What method would you have chosen? Why?
- Are there any methods you would avoid? Why?

1. Have you heard about **the coil**? (*If not, explain*)

*Probes:*

- What do you think about the coil?
- Is there anything which stops women from choosing to use the coil?
- How would you feel about having a coil fitted **immediately after delivery**?
- How would you feel about having a coil fitted **6 weeks after delivery**?
- What do you think are the advantages and disadvantages of these two options?

1. Have you heard about **the implant**? (*If not, explain*)

*Probes:*

- What do you think about the implant?
- Is there anything which stops women from choosing to use the implant?
- How would you feel about having an implant fitted **immediately after delivery**?
- How would you feel about having an implant fitted **6 weeks after delivery**?
- What do you think are the advantages and disadvantages of these two options?

1. How do you think we could make family planning services better for women like you, who have recently delivered?

*Probes:*

- How can we make sure everyone knows about the service?
- How can we make people feel comfortable accessing this service?
- How would you feel about discussing family planning during **antenatal visits**?
- How can we reassure people who have concerns about family planning?

1. Apart from your partner, who else influences your decisions on family planning? What do they think about the use of family planning after delivery?

*Probes:*

- Do they have any concerns about family planning after delivery?
- How could we address these concerns?

Closing comments

- “Is there anything else you would like to say before we end the interview?”
- *Thank the participant.*
- *Turn recorder off.*

## Interview schedule for male partners of postpartum women

**Version 1, 28^th^ April 2015 OXTREC Reference 565-15**

Participant Identification number: _____ Partner’s identification number: _____

Opportunity to ask questions and sign consent form

*Read out participant information sheet/get them to read it if literate.*

- Do you have any further questions?
- Would you like to talk to me about family planning?
- Do you understand and agree with everything I have said?

*If yes: Read consent form with participant and ask to sign or make a thumb print*

- Is it ok to start the interview now?
- I am going to turn on the tape recorder now and we can start.

Interview

1. Can you tell me a bit about yourself and your family?

*Probes:*

- Age, occupation, religion, education level, ethnic group
- Do you want to have another child?
  - If yes, how long would you like to wait?

1. Have you and your partner discussed family planning recently?

*Probes:*

- What did you discuss?
- When did you discuss this?
- What do you think she thinks about family planning? Is this the same or different to what you think about family planning?

1. Have you or your partner used family planning?

*Probes if yes:*

- What methods of family planning have you (or your partner) used?
- When did you use it?
- What was your experience using it?

*Probes if no:*

- Have you ever discussed family planning with a health worker?
- Have you ever thought of using family planning?
- What methods do you think might suit your needs?

1. Were you and your partner offered family planning after this delivery or after previous deliveries? Can you tell us your experience?

*Probes:*

- Where did your partner deliver? Were you present at or after the birth?
- Did you or your partner discuss family planning with your midwife or nurse?
- Did your partner attend post-natal clinic? Did you attend with her? What was your experience then?
- Did you speak to your VHT about family planning? Did you discuss it while your partner was pregnant or since delivery?

*Further probes* ***if offered*** *family planning:*

- What did you think about being offered family planning at this time?
- Did you decide to take up family planning? Why/why not?
- What method did you choose? Why?
- Are there any methods you would avoid? Why?

*Further probes* ***if not offered*** *family planning:*

- Would you have liked to discuss family planning with a health worker after your delivery? Why/why not?
- If family planning had been offered to you, do you think you would have taken it up? Why/why not?
- What method would you have chosen? Why?
- Are there any methods you would avoid? Why?

1. Have you heard about **the coil**? (*If not, explain*)

*Probes:*

- What do you think about the coil?
- Is there anything which stops women from choosing to use the coil?
- How would you feel about your partner having a coil fitted **immediately after delivery**?
- How would you feel about your partner having a coil fitted **6 weeks after delivery**?
- What do you think are the advantages and disadvantages of these two options?

1. Have you heard about **the implant**? (*If not, explain*)

*Probes:*

- What do you think about the implant?
- Is there anything which stops women from choosing to use the implant?
- How would you feel about your partner having an implant fitted **immediately after delivery**?
- How would you feel about your partner having an implant fitted **6 weeks after delivery**?
- What do you think are the advantages and disadvantages of these two options?

1. How do you think we could make family planning services better for families who have recently had a new baby?

*Probes:*

- How can we make sure everyone knows about the service?
- How can we make people feel comfortable accessing this service?
- How would you feel about discussing family planning during **antenatal visits**?
- How can we reassure people who have concerns about family planning?

1. Apart from your partner, who else influences your decisions on family planning? What do they think about the use of family planning after delivery?

*Probes:*

- Do they have any concerns about family planning after delivery?
- How could we address these concerns?

Closing comments

- “Is there anything else you would like to say before we end the interview?”
- *Thank the participant.*
- *Turn recorder off.*

## Interview Schedule for antenatal Women

**Version 1, 28^th^ April 2015 OXTREC Reference 565-15**

Participant Identification number: _____ Partner’s identification number: _____

Opportunity to ask questions and sign consent form

*Read out participant information sheet/get them to read it if literate.*

- Do you have any further questions?
- Would you like to talk to me about family planning?
- Do you understand and agree with everything I have said?

*If yes: Read consent form with participant and ask to sign or make a thumb print*

- Is it ok to start the interview now?
- I am going to turn on the tape recorder now and we can start.

Interview

1. Can you tell me a bit about yourself and your family?

*Probes:*

- Age, occupation, religion, education level, ethnic group
- Tell me about your partner. Married/unmarried, live together/apart, length of relationship, ethnic group of partner
- How many pregnancies have you had? How many children do you have? Have you lost any children?
- Do you want to have another child after this pregnancy?
  - If yes, how long would you like to wait?

1. Have you and your partner discussed family planning recently?

*Probes:*

- What did you discuss?
- When did you discuss this?
- What do you think he thinks about family planning? Is this the same or different to what you think about family planning?

1. Have you used family planning?

*Probes if yes:*

- What methods of family planning have you used?
- When did you use it?
- What was your experience using it?

*Probes if no:*

- Have you ever discussed family planning with a health worker?
- Have you ever thought of using family planning?
- What methods do you think might suit your needs?

1. Has someone discussed family planning with you during this pregnancy? Can you tell us your experience?

*Probes:*

- Have you discussed family planning with your midwife or nurse?
- Have you attended antenatal clinic? What was your experience then?
- Have you spoken to your VHT about family planning?

*Further probes* ***if offered*** *family planning:*

- What do you think about being offered family planning at this time?
- Have you decided to take up family planning after this pregnancy? Why/why not?
- What method will you choose? Why?
- Are there any methods you would avoid? Why?

*Further probes* ***if not offered*** *family planning:*

- Would you like to discuss family planning with a health worker in the antenatal clinic? Why/why not?
- If family planning were offered to you, do you think you would take it up? Why/why not?
- What method would you choose? Why?
- Are there any methods you would avoid? Why?

1. Have you heard about **the coil**? (*If not, explain*)

*Probes:*

- What do you think about the coil?
- Is there anything which stops women from choosing to use the coil?
- How would you feel about having a coil fitted **immediately after delivery**?
- How would you feel about having a coil fitted **6 weeks after delivery**?
- What do you think are the advantages and disadvantages of these two options?

1. Have you heard about **the implant**? (*If not, explain*)

*Probes:*

- What do you think about the implant?
- Is there anything which stops women from choosing to use the implant?
- How would you feel about having an implant fitted **immediately after delivery**?
- How would you feel about having an implant fitted **6 weeks after delivery**?
- What do you think are the advantages and disadvantages of these two options?

1. How do you think we could make family planning services better for women who have recently delivered?

*Probes:*

- How can we make sure everyone knows about the service?
- How can we make people feel comfortable accessing this service?
- How can we reassure people who have concerns about family planning?

1. Apart from your partner, who else influences your decisions on family planning? What do they think about the use of family planning after delivery?

*Probes:*

- Do they have any concerns about family planning after delivery?
- How could we address these concerns?

Closing comments

- “Is there anything else you would like to say before we end the interview?”
- *Thank the participant.*
- *Turn recorder off.*

## Interview Schedule for male partners attending antenatal clinic

**Version 1, 28^th^ April 2015 OXTREC Reference 565-15**

Participant Identification number: _____ Partner’s identification number: _____

Opportunity to ask questions and sign consent form

*Read out participant information sheet/get them to read it if literate.*

- Do you have any further questions?
- Would you like to talk to me about family planning?
- Do you understand and agree with everything I have said?

*If yes: Read consent form with participant and ask to sign or make a thumb print*

- Is it ok to start the interview now?
- I am going to turn on the tape recorder now and we can start.

Interview

1. Can you tell me a bit about yourself and your family?

*Probes:*

- Age, occupation, religion, education level, ethnic group
- Do you want to have another child after this one is born?
  - If yes, how long would you like to wait?

1. Have you and your partner discussed family planning recently?

*Probes:*

- What did you discuss?
- When did you discuss this?
- What do you think she thinks about family planning? Is this the same or different to what you think about family planning?

1. Have you or your partner used family planning?

*Probes if yes:*

- What methods of family planning have you (or your partner) used?
- When did you use it?
- What was your experience using it?

*Probes if no:*

- Have you ever discussed family planning with a health worker?
- Have you ever thought of using family planning?
- What methods do you think might suit your needs?

1. Has someone discussed family planning with you during this pregnancy? Can you tell us your experience?

*Probes:*

- Have you discussed family planning with a midwife or nurse?
- Have you attended antenatal clinic with your partner? What was your experience then?
- Have you spoken to your VHT about family planning?

*Further probes* ***if offered*** *family planning:*

- What did you think about being offered family planning at this time?
- Did you and your partner decide to take up family planning after the delivery? Why/why not?
- What method did you choose? Why?
- Are there any methods you would avoid? Why?

*Further probes* ***if not offered*** *family planning:*

- Would you have liked to discuss family planning with a health worker whilst your partner was pregnant? Why/why not?
- Do you think the nurse/midwife should discuss family planning during antenatal appointments? Why/why not?
- What method would you have chosen? Why?
- Are there any methods you would avoid? Why?

1. Have you heard about **the coil**? (*If not, explain*)

*Probes:*

- What do you think about the coil?
- Is there anything which stops women from choosing to use the coil?
- How would you feel about your partner having a coil fitted **immediately after delivery**?
- How would you feel about your partner having a coil fitted **6 weeks after delivery**?
- What do you think are the advantages and disadvantages of these two options?

1. Have you heard about **the implant**? (*If not, explain*)

*Probes:*

- What do you think about the implant?
- Is there anything which stops women from choosing to use the implant?
- How would you feel about your partner having an implant fitted **immediately after delivery**?
- How would you feel about your partner having an implant fitted **6 weeks after delivery**?
- What do you think are the advantages and disadvantages of these two options?

1. How do you think we could make family planning services better for families who have recently had a new baby?

*Probes:*

- How can we make sure everyone knows about the service?
- How can we make people feel comfortable accessing this service?
- How would you feel about discussing family planning during **antenatal visits**?
- How can we reassure people who have concerns about family planning?

1. Apart from your partner, who else influences your decisions on family planning? What do they think about the use of family planning after delivery?

*Probes:*

- Do they have any concerns about family planning after delivery?
- How could we address these concerns?

Closing comments

- “Is there anything else you would like to say before we end the interview?”
- *Thank the participant.*
- *Turn recorder off.*

## Focus group discussion guide

**Version 2, 25/6/2018**

[*Abbreviated: first part of discussion was about views on new health education films on family planning*].

**Questions about couples counselling (for women):**

How would you feel about discussing options for family planning to use after the delivery, together with your husband, during an antenatal clinic visit?

How would you feel about agreeing on a method of family planning to use after delivery, in advance with your husband / partner?

How would you prefer to do this? Prompts:

Alone with your partner

Together with a VHT

Together with a health worker, for example at the antenatal clinic

In some other way?

Why? (please explain the advantages and disadvantages for you of the different options)

Would you be able to come with your husband/partner to an antenatal clinic appointment?

What things might prevent you from doing this?

What might make it easier for you to do this?

**Questions about couples counselling (for men):**

How would you feel about discussing options for family planning to use after the delivery, together with your wife, during an antenatal clinic visit?

How would you feel about agreeing on a method of family planning to use after delivery, in advance with your wife / partner?

How would you prefer to do this? Prompts:

- Alone with your partner
- Together with a VHT
- Together with a health worker, for example at the antenatal clinic
- In some other way?

Why? (please, explain the advantages and disadvantages for you of the different options)

Would you be able to come with your wife/partner to an antenatal clinic appointment?

What things might prevent you from doing this?

What might make it easier for you to do this?

**Questions about couples counselling (for health workers):**

What do you think about the idea of asking men to come with their wives / partners to the antenatal clinic?

How would you feel about discussing options for family planning to use after the delivery, with couples, during an antenatal clinic visit?

What things might prevent you from doing this?

Probes: Would you have enough time to do this?

Do you have enough knowledge to do this?

What might make it easier for you to do this?

Would it be possible to combine this with couples’ counselling on antenatal testing for HIV?

**Closing comments**

- “Is there anything else you would like to say before we end the interview?”
- *Thank the participants.*
- *Turn recorder off.*
